# Effects of Nitrogen and Intercropping on the Occurrence of Wheat Powdery Mildew and Stripe Rust and the Relationship With Crop Yield

**DOI:** 10.3389/fpls.2021.637393

**Published:** 2021-02-24

**Authors:** Chaosheng Luo, Liankun Ma, Jinhui Zhu, Zengpeng Guo, Kun Dong, Yan Dong

**Affiliations:** ^1^College of Resources and Environment, Yunnan Agricultural University, Kunming, China; ^2^School of Life Sciences, Lanzhou University, Lanzhou, China; ^3^College of Animal Science and Technology, Yunnan Agricultural University, Kunming, China

**Keywords:** N level, powdery mildew, stripe rust, intercropping, wheat disease and yield

## Abstract

Wheat powdery mildew (*Blumeria graminis* f. sp. *tritici*) and stripe rust (*Puccinia striiformis* Westend f. sp. *tritici*) restrict wheat production in southwest China. Nitrogen fertilizers may influence outbreaks of these wheat diseases where wheat/faba beans are intercropped. To clarify how intercropping and varying nitrogen levels influence wheat powdery mildew and stripe rust and their relationship with crop yield, two consecutive field experiments were conducted from 2015 to 2017. Three cropping regimens (monocropped wheat, monocropped faba beans, and intercropped wheat/faba beans) and four nitrogen levels [N0 (0 kg⋅ha^–1^), N1 (90 kg⋅ha^–1^), N2 (180 kg⋅ha^–1^), and N3 (270 kg⋅ha^–1^)] were evaluated. In two consecutive planting seasons, the incidence and disease index of powdery mildew and stripe rust increased, while the disease index was more affected by nitrogen levels than their incidence. Both diseases were most prevalent at the N3 level. Compared with monocropping, intercropping (N0–N3 levels) reduced the incidence of powdery mildew by 2.8–37.0% and disease index by 15.5–47.4%, increased the relative control effect by 10.7–56.2 and 16.3–47.2%, reduced the incidence of stripe rust by 2.9–42.7% and disease index by 8.3–42.2%, and increased the relative control effect by 5.9–43.7 and 8.8–42.1%. The relative control efficacy of intercropping was most affected by N2 level. Intercropping yield increased with increasing nitrogen by 25.0–46.8%, and overall land equivalent ratio (LER) was 1.30–1.39. The correlation coefficient between disease index and wheat yield for both diseases was −0.7429 to −0.9942, a significant negative correlation, most significant at N1. Nitrogen regulation in intercropped wheat/faba beans can control powdery mildew and stripe rust, and optimize wheat yield. Intercropping at 180 kg ha^–1^ N2 resulted in the highest yield.

## Introduction

Wheat (*Triticum aestivum* L.) is a staple food for a large percentage of the global population. Powdery mildew of wheat and stripe rust is caused by the biotrophic fungi *Blumeria graminis* f. sp. *tritici* and *Puccinia striiformis* Westend f. sp. *tritici* Erikss, respectively. These diseases reduce wheat yields worldwide. In China, the mean area of wheat affected annually by powdery mildew is approximately 6.85 million ha and approximately 4.2 million ha by stripe rust (Zhao, 2004; Tosti and Guiducci, 2010; Jiang et al., 2017). Both diseases can have serious consequences and even the wheat yield of Yunnan Province has been reduced by 10–50% by both diseases (Huo et al., 2002; He et al., 2011; Zhang et al., 2016).

The wheat powdery mildew in Yunnan caused huge losses in local yield and caused severe harm to other surrounding areas (Sheng and Duan, 1996). The pathogen that causes wheat stripe rust has widespread transmission patterns similar to that of powdery mildew (Huang et al., 2018). The methods of control, including enhancing host resistance and by chemical means, have been widely adopted. However, due to the continuous selection of populations of the pathogen and the emergence of new virulent strains, it continues to persist despite the continuous development of different wheat varieties. Chemical control causes environmental pollution and the accumulation of pesticide residues, which endangers human health. Therefore, it is of considerable benefit to select cropping regimens and field management measures that optimally control diseases in crops.

Intercropping is a mode of planting that has contributed considerably to the success of both traditional and modern agriculture, having the advantages of providing a natural barrier to disease and increasing biodiversity (Lamondia et al., 2002; Gao et al., 2010; Lopes et al., 2016). Similarly, Zhu et al. (2000) have reported that mixed planting of disease-susceptible and resistant rice varieties reduces the occurrence of rice blast, resulting in increased rice yield. Intercropping wheat with other crop species has been reported to reduce the damage caused by powdery mildew and stripe rust (Cao et al., 2015). Others have reported similar results, with intercropping boosting yield, reducing disease prevalence, and enhancing ecosystem stability (Li et al., 2009; Su et al., 2013). Nitrogen (N) is not only an important nutritional factor that promotes crop growth and increases crop yield but is also known to impact stripe rust and powdery mildew infection and disease severity directly (Ash and Brown, 1991; Chen et al., 2007; Devadasa et al., 2014; Zhu et al., 2017a). These results are attributed to the increased density of canopy resulting from the application of nitrogen fertilizer, providing a favorable microclimate for the development and spread of pathogenic fungi (Darwinkel, 1980; Danial and Parlevliet, 1995; He, 2009). Other studies have suggested that the effects of nitrogen on pathogenic fungi are mediated via increased nitrogen content of the host tissue by acting as a substrate for pathogen growth (Chen et al., 2013; Zhu et al., 2017a).

Wheat and faba bean (*Vicia faba* L.) intercropping is not only a common planting pattern in Yunnan Province, China, but also widely adopted as a typical intercropping pattern of cereal and legumes in many other countries and regions (Chen et al., 2007; Bargaz et al., 2015; Tosti et al., 2016). Previous studies have demonstrated that faba bean and wheat intercropping reduces yield losses associated with wheat powdery mildew and stripe rust (Yang et al., 2009; Jiang et al., 2012; Xiao et al., 2018). We have also found that the rational use of nitrogen fertilizer is among the key factors that control wheat powdery mildew and stripe rust and therefore can increase yields in such intercropping systems (Chen et al., 2013; Yang et al., 2013; Zhu et al., 2017a). However, there are relatively few reports of the effects of both nitrogen level and intercropping patterns on the occurrence of wheat powdery mildew and stripe rust and their relationship with yield. Therefore, through two continuous planting seasons field experiments of wheat and faba bean intercropping using different levels were conducted. The purpose of the study was (1) to explore the effects of nitrogen regulation and wheat/faba bean intercropping on the occurrence of wheat powdery mildew and stripe rust and their relative control efficacy. (2) Determine the relationship between the incidence and severity of disease and yield, so as to provide theoretical guidance for the optimization of nitrogen fertilizer application in intercropping systems.

## Materials and Methods

### Experimental Site and Cultivars

The 2-year field experiment (2015–2016 and 2016–2017) was established at the E Feng village in E Shan County (102°24′E, 24°11′N, altitude: 1691 m) in Yuxi, Yunnan Province, China, located 28 km to the southwest of Yuxi city. The site experiences mean annual temperature and rainfall of approximately 16.3°C and 1120 mm, respectively. Chives had been cultivated in the field during the year previous to the establishment of the present study. The growth period for wheat is 185 days, while faba beans are harvested earlier. The symbiotic period of wheat and faba beans is approximately 175 days. The mean monthly temperature and rainfall during the crop growing season are displayed in Table 1.

**TABLE 1:** Mean monthly temperatures and rainfall during the crop growing season in 2015–2016 and 2016–2017.

Month		October	November	December	January	February	March	April
Temperature (°C)	2015–2016	17.5	15.1	10.7	9.0	10.2	15.7	19.0
	2016–2017	19.2	14.3	11.8	11.9	12.6	14.6	17.4
Rainfall (mm)	2015–2016	124.3	68.5	10.2	17.3	23.6	26.2	37.7
	2016–2017	117.8	74.3	13.4	18.9	25.8	30.3	45.2

The site had paddy-type soil whose basic physical and chemical properties were organic matter: 28.9 g⋅kg^–1^, total nitrogen: 2.2 g⋅kg^–1^, total phosphorus: 0.8 g⋅kg^–1^, total potassium: 18.3 g⋅kg^–1^, alkali hydrolyzable nitrogen: 102.0 mg⋅kg^–1^, available phosphorus: 36.9 mg⋅kg^–1^, available potassium: 100.5 mg⋅kg^–1^, and pH 6.7.

Test varieties: *V. faba* L. *cv.* “Yuxi dalidou” and *T. aestivum* L. *cv.* “Yunmai-52” were investigated in the present study. Both cultivars were purchased from the Institute of Food Crops, Yunnan Academy of Agricultural Sciences.

### Experimental Design and Crop Management

The experiment had a two-factor design (termed A and B), where factor A was the planting pattern, which consisted of wheat monocropping, faba bean monocropping, and wheat/faba bean intercropping. Factor B represented nitrogen application level (wheat nitrogen fertilizer rate), used at four concentrations, termed N0-N3 (0, 90, 180, and 270 kg⋅ha^–1^). The quantity of nitrogen fertilizer applied at each level for faba beans was half of that of wheat, namely 0, 45, 90, and 135 kg ha^–1^, respectively. The totals included 12 treatments, and the experiment was conducted in a completely randomized block design with three replications. A ridge 0.5 m wide was placed between adjacent blocks, while blocks were separated by 1.0 m wide ridges. Each plot size was 6.0 m long by 5.4 m wide (6.0 m × 5.4 m = 32.4 m^2^).

The fertilizers tested were urea (N: 46%), superphosphate (P: 7%), and potassium sulfate (K: 41%). The phosphate and potassium fertilizers were both applied at 90 kg ha^–1^ and used once as base fertilizers. Nitrogen fertilizer was applied as a base fertilizer once in both the monocropping and intercropping of faba beans prior to sowing. For wheat, half of the nitrogen fertilizer was applied prior to sowing, the remaining half at the jointing stage of wheat. The daily management of other fields was consistent with local routines.

**FIGURE 1:**
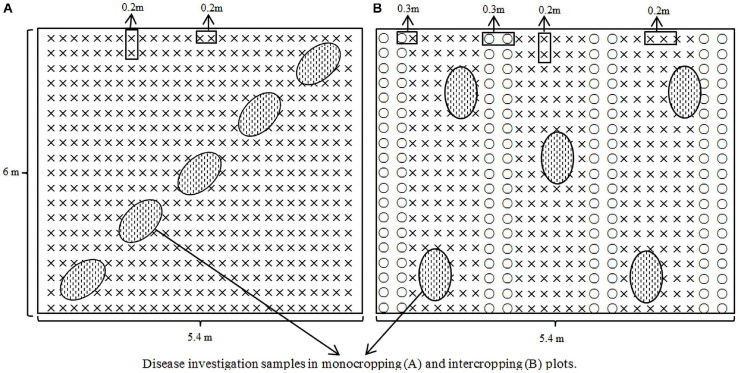
Illustration of planting patterns and sampling in the field experiments: **(A)** monocropped wheat plot, **(B)** intercropped wheat with faba beans (× representing wheat, ○ representing faba beans).

Wheat and faba beans were sown at the same time, in October 2015 and October 2016, and harvested approximately 160 days post-emergence, in April the following year. The monocropping and intercropping of faba beans was accomplished with on-demand sowing (row spacing: 0.3 m; plant spacing: 0.15 m). The planting patterns were as follows: monocropping of faba beans (18 rows/plot), intercropping of faba beans (6 rows/plot), monocropping, and intercropping of wheat (row spacing: 0.2 m; sowing rate: 18 g/row), monocropping of wheat (27 rows/plot), and intercropping of wheat (18 rows/plot). In the intercropped plots, the faba beans and wheat were planted in a 2:6 row ratio, that is, two rows of faba beans intercropped with six rows of wheat. In the intercropped plot, there were three wheat planting belts and two faba bean planting belts (Figure 1). The planting methods, plant spacing, row spacing, and quantities of fertilizers in the monocropped and intercropped wheat were identical to that of the faba beans in the intercropped plot.

### Wheat Disease Investigation Methods

Infection by wheat powdery mildew and stripe rust occurred naturally in the field. No fungicides were used to control the outbreaks during the growing season. The disease was evaluated during the filling period. When investigating monocropped wheat, five locations in each plot were selected using a diagonal method, with 10 stalks investigated at each point, for a total of 50 stalks. Five locations were also selected for the edge and inner rows of the intercropped wheat (Figure 1), of which 10 stalks were selected for each of the side and inner rows. All green leaves on the selected stems were investigated in both monocropped and intercropped plots, a total of 190–230 leaves in each plot.

Wheat powdery mildew and stripe rust was investigated at the same plot at the same time, and the incidence and severity of the two diseases recorded at the grain filling stage of wheat for each plot. The severity of disease was evaluated as grades 1-9 (ICARDA, 1986). Grade 0 indicated no symptoms of visible spore spots on the wheat leaves; grade 1 indicated spore spots covering ≤5% of the total leaf area; grade 3 indicated spore spots on 6–25% of the total leaf area; grade 5 indicated spore spots on 26–50% of the total leaf area; grade 7 indicated spore spots on 51–75% of the total leaf area and grade 9 indicated extensive spore spots on leaves and stems (≥76%), multiple leaves having died. These data were used to calculate disease incidence, disease index, and relative control efficacy for each plot, as follows:

$$\begin{array}{l} \text{Incidence }(\%)=\text{number of damaged leaves}\\ \;/\text{total number of leaves }\times \;100\% \end{array}$$

$$\begin{array}{l} \text{Disease index}=\sum^{\text{​}}(\text{number of diseased eaves in each scale}\\ \;\times \text{ value of the corresponding scale})\\ \;/(\text{total leaf number scored}\times 9)\times 100 \end{array}$$

$$\mathrm{Relative}\,\mathrm{control}\,\mathrm{efficacy}=\left[\,\left(D_{M}-D_{I}\right)/D_{M}\right]\times 100\%$$

where *D* represents the incidence and disease index, *M* and *I* represent monocropping and intercropping, respectively.

### Yields and Land Equivalent Ratio

The wheat and faba beans were reaped by hand when fully mature in mid April 2016 and 2017. An area of approximately 7.2 m^2^ of wheat was harvested from the monocropped wheat plots. An area of 3.6m^2^ representing approximately 120 faba bean plants was harvested from the monocropped faba bean plots. To avoid the influence of border effects, in the monocropped plots, after removal of the 3 outermost side rows, 6 rows of wheat or faba beans were harvested, respectively, and in the intercropped plots, the two rows of faba beans and six rows of wheat in the middle of the plot were harvested. The wheat and faba beans were dried naturally after harvesting, to allow the gain in grain weight to be measured. The weighed grain total yield was converted into grain yield per unit area.

The land equivalent ratio (LER) was calculated to evaluate the advantage of intercropping at various N levels using the following formula:

$$\text{LER}=\frac{Y_{i\,w}}{Y_{m\,w}}+\frac{Y_{i\,f}}{Y_{m\,f}}$$

where *Y*_*iw*_ and *Y*_*mw*_ represent the yield of wheat in intercropping and monocropping regimens, respectively, and *Y*_*if*_ and *Y*_*mf*_ are the yield of faba beans in intercropped and monocropped plots, respectively. LER > 1.0 indicates that intercropping provided greater yield than the corresponding monocropping, and vice versa (Willey, 2007).

### Statistical Analysis

Statistical analysis software (SPSS, 2008 version 19.0, SPSS Inc., Chicago, IL, United States) was used to analyze the data. The significance of the treatments was tested by two-factor design analysis. The differences between treatments were compared by SPSS software for analysis of variance (ANOVA), and the mean value compared using the Tukey HSD test at the 5% level (α = 0.05).

## Results

### Effect of N Levels and Intercropping on Wheat Disease Development

#### Powdery Mildew Disease Development

**FIGURE 2:**
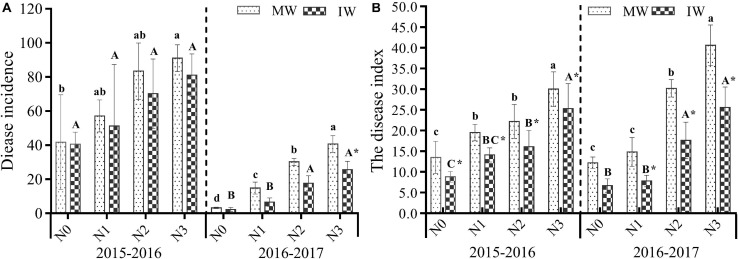
Incidence of disease **(A)** and the disease index **(B)** for powdery mildew on monocropped and intercropped wheat at different N levels in 2015–2017. MW: monocropped wheat; IW: intercropped wheat. The different capital letters and small letters represent significant differences between different N levels in the same planting regimens at *P* < 0.05 levels. *Represents significant difference between monocropped and intercropped regimens at the same N level (*P* < 0.05).

In the 2-year experiment, it was found that the incidence and disease index of powdery mildew in monocropped and intercropped wheat displayed an increasing trend as the levels of N increased (Figure 2 and Table 2). Disease levels were at their greatest in plants at the N3 level. Compared with N0, the N1–N3 treatments increased the incidence of wheat powdery mildew in monocropped and intercropped plants by 37.1–118.6% and 26.5–100.0%, respectively (2015–2016), and 363.5–1168.8 and 184.3–995.7%, respectively (2016–2017). Compared with N0, N1–N3 treatments increased the disease index of wheat powdery mildew in monocropped and intercropped plots by 44.3–122.0 and 59.5–186.7%, respectively (2015–2016); 21.6–232.8 and 17.0–283.5%, respectively (2016–2017). The results demonstrated that increasing the quantity of applied nitrogen exacerbated the occurrence of wheat powdery mildew, and the effects of nitrogen fertilizer application on disease index was greater than the incidence of wheat powdery mildew.

**TABLE 2:** Effects of N levels and planting pattern on wheat yield, powdery mildew and stripe rust.

Influence factors	2015–2016					2016–2017				
	Powdery mildew		Stripe rust			Powdery mildew		Stripe rust		
	Disease	Disease	Disease	Disease	Yield	Disease	Disease	Disease	Disease	Yield
	incidence	index	incidence	index		incidence	index	incidence	index	
N levels	1.3*ns*	11.0**	4.7*	20.5**	52.1**	60.5**	73.4**	93.0**	66.0**	7.7**
Plant patterns	9.8**	19.4**	0.2*ns*	11.9**	43.7**	11.1**	9.3**	44.5**	53.9**	52.3**
Plant patterns × N levels	0.1*ns*	0.6*ns*	0.6*ns*	0.1*ns*	2.0*ns*	2.3*ns*	1.5*ns*	2.1*ns*	2.7*ns*	0.3*ns*

The data in the table are *F*-values of the interaction between N levels and planting patterns (two-way ANOVA, *P* < 0.05). *Represents significant difference (*P* < 0.05), **represents significant difference (*P* < 0.01), ns represents no significant difference.

Compared with monocropping, intercropping reduced the incidence and disease index of wheat powdery mildew caused by application of nitrogen. However, there was a significant difference in the disease index between intercropping and monocropping (except for the 2016–2017 N0 level) (*P <* 0.05), while intercropping did not significantly reduce the disease incidence of powdery mildew. For the N0–N3 levels intercropping for 2 years reduced the incidence of wheat powdery mildew by 2.9–18.7% and 27.1–55.3%, in the two planting seasons, respectively, reducing the disease index of wheat powdery mildew by 15.5–34.6 and 37.0–123.6%, in the two planting seasons respectively. The results indicated that intercropping effectively reduced the severity of wheat powdery mildew but was relatively poor at reducing its incidence.

To further evaluate the overall effects of nitrogen level and mode of intercropping on the occurrence of disease and its control effect, it can be seen from Table 2 that there was no significant interaction between plant patterns and N levels. The control effect of 2-year intercropping wheat on powdery mildew was 10.7–19.4 and 29.2–56.2% (based on incidence), for which the N2 level was relatively superior. The corresponding values were 16.3–32.8 and 37.3–47.2% when based on disease index (Table 3), for which the N0 level scored well, relatively, in control effect.

**TABLE 3:** Relative control efficacy (%) for wheat powdery mildew and stripe rust at different N levels from 2015 to 2017.

N level	2015–2016				2016–2017			
	Powdery mildew		Stripe rust		Powdery mildew		Stripe rust	
	Incidence	Disease index	Incidence	Disease index	Incidence	Disease index	Incidence	Disease index
N0	10.7 ± 16.8ab	32.8 ± 9.4a	43.2 ± 6.6a	41.2 ± 11.6a	29.2 ± 5.4c	45.8 ± 7.5a	5.9 ± 8.2c	8.8 ± 5.8b
N1	15.3 ± 15.1a	27.6 ± 4.9ab	43.7 ± 18.2a	42.1 ± 1.4a	56.2 ± 5.9a	47.2 ± 3.1a	15.8 ± 5.8b	20.5 ± 4.5a
N2	19.4 ± 10.3a	28.2 ± 5.9ab	30.7 ± 25.5ab	16.8 ± 1.7b	42.3 ± 11.5ab	42.3 ± 11.8a	22.9 ± 10.6ab	18.4 ± 4.2ab
N3	11.4 ± 7.0ab	16.3 ± 10.2b	27.2 ± 17.2b	16.6 ± 10.7b	37.3 ± 6.5bc	37.3 ± 6.5a	26.2 ± 12.0a	23.6 ± 9.1a
Mean	14.2	26.2	36.2	29.2	41.3	43.1	17.7	17.8

*Small letters represent significant differences between different N levels at *p* < 0.05.*

#### Stripe Rust Disease Development

**FIGURE 3:**
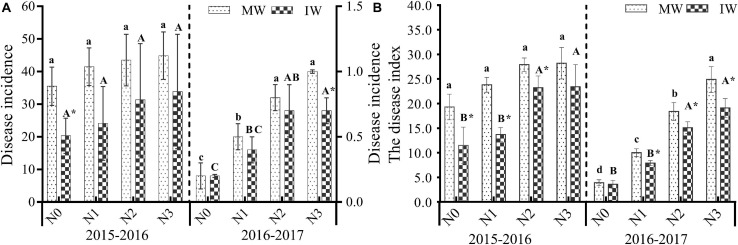
Disease incidence **(A)** and disease index **(B)** of stripe rust in monocropped and intercropped wheat at different N levels in 2015–2017. MW: monocropped wheat; IW: intercropped wheat. The different capital letters and small letters represent significant differences between different N levels in the same planting regimens at *P* < 0.05 levels. *Represents significant difference between monocropped and intercropped regimens at the same N level (*P* < 0.05).

After the 2-year experiment, it was found that the incidence and disease index of stripe rust in monocropped and intercropped wheat displayed an increasing trend as the levels of N increased (Figure 3 and Table 2). At the N3 level, wheat stripe rust disease was the most serious. Compared with N0, the N1–N3 treatments increased the incidence of wheat stripe rust in monocropped and intercropped plants by 17.0–26.6 and 18.6–66.9%, respectively (2015–2016), and 143.2–395.5 and 98.1–256.2%, respectively (2016–2017). Compared with N0, N1–N3 treatments increased the disease index of wheat stripe rust in monocropped and intercropped plots by 22.9–45.9 and 19.2–102.7%, respectively (2015–2016), and 152.6–530.8 and 118.6–428.5%, respectively (2016–2017). The results demonstrate that increasing the quantity of applied nitrogen exacerbated the occurrence of wheat stripe rust, while the disease index was more affected by the application of nitrogen fertilizer than the incidence of wheat powdery mildew.

Compared with monocropping, intercropping at each N level reduced the incidence and disease index of wheat stripe rust. There was a significant difference in the disease index of monocropped and intercropped wheat at the various N levels (except for the 2015–2016 N3 level and 2016–2017 N0 level (*P <* 0.05). Only the incidence in 2015–2016 at the N0 level and in 2016–2017 at the N3 level were significantly different (*P <* 0.05). For the N0–N3 levels intercropping for 2 years reduced the incidence of wheat stripe rust by 32.4–74.6 and 2.7–35.2% for the two planting seasons respectively, reducing the disease index of wheat stripe rust by 20.7–70.9 and 9.1–30.2% in the two planting seasons respectively. The results showed that intercropping was effective in controlling the severity of wheat stripe rust, but relatively weak in controlling the incidence of wheat powdery mildew.

To further evaluate the overall effects of N levels and plant patterns on the occurrence of disease and its control effect, it can be seen from Table 2 that there was no significant interaction between plant patterns × N levels, but both had significant effects on disease severity. The control effect of 2-year intercropping wheat on stripe rust was 27.2–43.7 and 5.9–26.2% (based on incidence), for which the N2 level was relatively good. The corresponding values were 16.6–42.1 and 8.8–23.6% based on disease index (Table 3), for which the N1 level scored well, relatively, in control effect.

### Effects of Planting Patterns and N Levels on Wheat Yield

#### Yields and LER

As shown in Table 4, the grain yield of monocropped and intercropped wheat displayed an initially increasing then decreasing trend at increasing levels of N fertilizer, reaching a maximum with the N2 level. Thus, the level of nitrogen and planting pattern significantly affected yield (Table 2). Compared with N0, from 2015 to 2016, the level of N (N0–N3) had a significant impact on the grain yield of monocropped and intercropped wheat (*P <* 0.05), although monocropped wheat was not significantly different between N1 and N2. The use of N fertilizer increased the yield of monocropped wheat by 23.4–44.4%, and intercropped wheat by 7.5–26.8%. From 2016 to 2017, the level of N (N0–N3) had a significant impact on the grain yield of intercropped wheat, but no significant impact on monocropped wheat (except for the N2 level), the corresponding yield increasing by 22.3–40.0% for monocropped wheat, and 6.4–26.1% for intercropped wheat.

**TABLE 4:** Yield (×10^3^ kg⋅ha^–1^) and LER of monocropped and intercropped wheat at different N levels 2015–2017.

N Level	2015–2016			2016–2017			LER	
	MW	IW	Increase	MW	IW	Increase	2015-2016	2016-2017
N0	3.41 ± 0.25c	4.92 ± 0.28c*	44.35%	3.33 ± 0.62a	4.89 ± 0.45b*	46.83%	1.37 ± 0.04	1.42 ± 0.17
N1	4.64 ± 0.23ab	5.73 ± 0.23b*	23.39%	4.24 ± 0.54a	5.88 ± 0.35ab *	38.61%	1.28 ± 0.03	1.41 ± 0.09
N2	4.81 ± 0.12a	6.24 ± 0.20a*	29.66%	4.67 ± 0.66a	6.17 ± 0.34a *	32.26%	1.35 ± 0.02	1.34 ± 0.08
N3	4.23 ± 0.21b	5.29 ± 0.56bc*	24.95%	4.08 ± 0.29a	5.21 ± 0.56ab *	27.66%	1.29 ± 0.02	1.32 ± 0.02
Mean	4.27	5.54*	30.59%	4.08	5.54*	36.34%	1.32	1.37

MW: monocropped wheat; IW: intercropped wheat. The different capital letters and small letters represent significant differences between different N levels in the same planting regimens at *P* < 0.05 levels. *Represents a significant difference between monocropped and intercropped regimens at the same N level (*P* < 0.05).

Compared with monocropping, from 2015 to 2016, intercropped wheat yield increased by 23.4–44.4%, mean yield increasing by 30.6%, with LER values of 1.37, 1.28, 1.35, and 1.29 at the N0–N3 levels, respectively, and a mean of 1.32. For 2016–2017, intercropped wheat yield increased by 27.7–46.8%, the mean yield increasing by 36.3%, with LER values of 1.42, 1.41, 1.34, and 1.32 at the N0–N3 levels, respectively, with a mean of 1.37. The 2-year test results demonstrated that wheat intercropped at the N2 level of supplementation displayed the highest grain yield. The LER of each nitrogen application level was greater than 1, indicating that wheat and faba bean intercropping displayed a significant increase in yield, but that advantage decreased as nitrogen was increasingly applied.

#### Correlation Between Disease Index and Yield of Wheat

**FIGURE 4:**
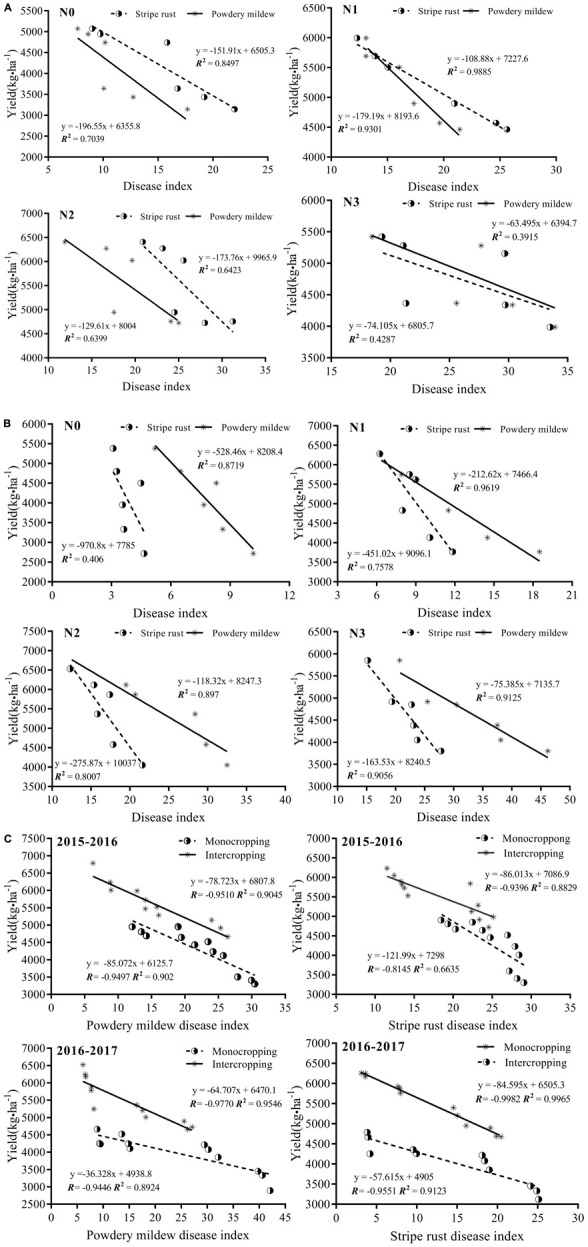
Correlation between disease index and yield at different N levels from 2015 to 2017. **(A)** Correlation between disease index and yield of wheat for different levels of applied N in 2015–2016. **(B)** Correlation between disease index and yield of wheat for different levels of applied N in 2016–2017. **(C)** Correlation between disease index and yield of mono-and intercropped regimens, 2015–2017.

Using linear regression analysis between disease index and yield for the two diseases of wheat, the correlation coefficient (*R-*value) between the disease index and yield was found to be >0.6 for different treatments (Figure 4), demonstrating a strong negative correlation, i.e., as the disease index increased, the yield of wheat decreased. From 2015 to 2016, with increased supplementation of N, the *R*^2^ values of wheat powdery mildew and rust both initially increased then decreased. The *R*^2^ values of rust were both greater than powdery mildew. The *R*^2^ values for the two diseases of wheat were N1 > N0 > N2 > N3. From 2016 to 2017, as nitrogen supplementation increased, the *R*^2^ values of wheat rust and powdery mildew first increased then decreased. The *R*^2^ values of powdery mildew were both greater than those of rust. The *R*^2^ values of both diseases were N1 > N2 > N3 > N0. The results above indicate that when the nitrogen level was N1 or N2, combined with the intercropping regimen, the measures controlled the occurrence of both diseases, and the recovery of yield loss was greater.

The results of the 2-year test indicated that the correlation between disease index and yield of intercropped wheat were higher than those of the monocropped crop, indicating that the intercropping planting pattern was superior for recovery of yield loss. In general terms, intercropping measures with a selection of N1 or N2 can recover yield loss to a certain extent, which provides a theoretical basis for nitrogen regulation and intercropping for controlling the occurrence of disease and increasing crop yield.

## Discussion

### Effect of N Level on the Occurrence of Wheat Powdery Mildew and Stripe Rust

An inappropriate application of nitrogen fertilizer is a principal reason for the frequent occurrence of crop diseases and the decrease of yield. Previous studies have demonstrated that as applied nitrogen has been increased, the severity of wheat leaf fungal disease has increased, its severity greatest at high nitrogen levels (Danial and Parlevliet, 1995; Olesen et al., 2003; Chen et al., 2013; Zhu et al., 2017a), consistent with the results of the present study. The mechanisms by which nitrogen increases foliar fungal disease severity can be summarized as follows: (1) the increased concentration of foliar nitrogen becomes a resource for the pathogen, with higher nitrogen levels in leaves providing nutrients for growth and reproduction of pathogenic fungi (Jensen and Munk, 1997; Strengbom et al., 2002); (2) the production of defensive compounds in the plant decrease, allowing the pathogenic fungi to more easily infect the plants (Sander and Heitefuss, 1998; Jin et al., 2008); and (3) the microclimate within the canopy is of a higher humidity with reduced permeability, more suitable for infection by and dissemination of pathogenic fungi (Jenkyn, 1976; He, 2009). In the 2-year field experiment, the severity of wheat powdery mildew and stripe rust strongly increased as higher levels of nitrogen were applied. For the two planting seasons, powdery mildew increased by a mean of 77.0–134.1 and 109.4–154.7%, and stripe rust by 37.8–350.2 and 74.4–287.8%, respectively. The incidence and disease index of wheat powdery mildew and stripe rust were the highest at the N3 level, followed by N2, N1, and N0. These results are similar to those of Qiao et al. (2010) and Zhu et al. (2020) using a wheat/faba bean system. Reducing the quantity of applied nitrogen fertilizer is beneficial for regulating air-borne diseases in wheat. The incidence of the two diseases in the second year was lower than that in the first year, indicating that the occurrence of the disease was related to conditions for colonization and spore transmission in the current season (Guo et al., 2020). There was a positive correlation between the disease index of powdery mildew and stripe rust with nitrogen levels, disease severity greater at high nitrogen levels in monocropped and intercropped wheat (Figure 4). That implies that management of nitrogen fertilization may be the factor key to the control of wheat powdery mildew and stripe rust in monocropping and intercropping. In addition, seasonal variations of disease severity are dependent on climatic factors of the current season, so the incidence of the disease is not exactly the same in any 2 years.

### Effect of Intercropping on Control of Wheat Disease

Previous studies have demonstrated that intercropping is an effective method of controlling crop diseases (Zhu et al., 2000, 2017b; Ning et al., 2017). In this 2-year field experiment, the mean relative control effect for wheat/faba bean intercropping against powdery mildew was 14.2%, 43.1% (2015–2016) and 26.2%, 43.1% (2016–2017), respectively for the N0–N3 levels. The mean relative control effect against stripe rust was 36.2%, 29.2% (2015–2016) and 17.7%, 17.8% (2016–2017) (Table 4). These results are consistent with the studies of Xiao et al. (2005) and Zhu et al. (2017a), although in wheat/faba bean intercropping systems, they found that the incidence of stripe rust and powdery mildew were both substantially lower.

Infection by wheat powdery mildew and stripe rust pathogens were affected by canopy microclimate temperature, humidity, and light. High temperature and humidity represents an environment preferred by infecting pathogens (Li et al., 2013). Conditions in which wheat planting density is high and canopy ventilation poor with a long duration of rainfall is those that lead to serious disease outbreaks, especially in single planting patterns. At this experimental site, faba beans are taller than wheat, so the canopy in wheat/faba bean intercropping is different from that of wheat monocropping. This allows stronger sunlight with better air circulation, combined with lower temperature and humidity in the wheat canopy, which is less conducive to infection by pathogens (Guo et al., 2020). The more favorable field canopy microclimate of wheat and faba bean intercropping is an important rationale for the control wheat powdery mildew and stripe rust. This related to the role of faba beans as a physical barrier that blocks the transmission of pathogens across the planted area (Lopes et al., 2016). Intercropping has a strong control effect on highly specialized pathogens, especially wheat and rice diseases (Mundt and Leonard, 1985). Zhu et al. (2017a), suggesting that the reason for wheat/faba bean intercropping systems reducing the incidence of wheat powdery mildew at high nitrogen levels was the promotion of nitrogen transport from wheat leaves to grains in intercropping, preventing a reduction in substances that defend plant health caused by excessive nitrogen accumulation in leaves, maintaining the exuberant growth of wheat, and improving disease resistance.

In the 2-year field experiment, we found that the relative control effects of intercropping on wheat powdery mildew and stripe rust were different for different levels of applied nitrogen. The relative control effect at the N2 and N3 levels was lower than those of N0 and N1 (Table 4), in which the incidence and disease index of powdery mildew were significantly lower than for N2 and N3. This is related to the deficiency of nitrogen nutrition in monocropping wheat caused by lower nitrogen fertilizer input, which leads to a decrease in wheat resistance to powdery mildew. In wheat/faba bean intercropping systems, faba beans have the role of biological nitrogen fixation, allowing the transfer and utilization of fixed nitrogen to wheat, improving wheat nitrogen nutrition and alleviating the symptoms of wheat nitrogen deficiency, resulting in a reduction in the occurrence and degree of harm of powdery mildew (Fu et al., 2016; Li, 2016). The incidence and disease index of stripe rust exhibited the same trend as powdery mildew (Figures 2, 3), suggesting that the disease control efficacy of intercropping was related to the severity of the disease. The incidence and disease index of both diseases increased at high nitrogen levels in wheat monoculture and wheat/faba bean intercropping (Figures 2, 3). Therefore, the nitrogen level not only affected the occurrence of disease but also regulated the relative control efficacy of the intercropping system.

### Effects of Nitrogen Level and Intercropping on Yield Advantage

Previous studies have demonstrated that intercropping is beneficial to yield and LER over monoculture (Tosti and Guiducci, 2010; Yu et al., 2015). A meta-analysis demonstrated that the global intercropping LER was 1.30 (Martin-Guay et al., 2018). In this 2-year field experiment, the results indicated that wheat and broad bean intercropping contributed to wheat yield. For four nitrogen application levels, the mean yield increased by 30.6 and 36.3% for the first and second seasons, respectively (Table 2). LER is an important index for the measurement of the efficiency of land use. The LER of wheat/faba bean intercropping was 1.30–1.39 (Table 2). Wheat and faba bean intercropping is a typical regimen for cereals and legumes, as there is a mutually beneficial relationship between the two. Leguminous crops fix nitrogen through biological mechanisms, and cereals can use more nitrogen in the field. Thus, the growth of cereal crops is superior in intercropping systems, with higher yields (Fu et al., 2016; Chai et al., 2017). Chen et al. (2013) found that the intercropping of wheat and faba beans improved the nitrogenous nutrition of wheat where there was a deficiency of nitrogen in the field. Intercropping can also change the layout and canopy structure of the crops and improve the efficiency of light radiation utilization and canopy air circulation. Rice plants are higher than spinach because rice is a high-stem crop. Ning et al. (2017) found that compared with water spinach, rice had more space to grow and therefore is able to exploit this competitive advantage. In addition to the factors described above, intercropping can continue to control disease and reduce yield loss caused by the disease, an important explanation for the increase in yield due to intercropping. Wheat interplanted with faba beans in the highlands of Ethiopia increased yields, reduced weeds and the pressure of disease, improving land-use efficiency (Agegnehu et al., 2008). Rice/arrowhead (*Sagittaria sagittifolia*) and rice-*Oenanthe javanica* intercropping is not only effective in reducing the occurrence of the diseases of rice but has also been shown to increase crop yield and land-use efficiency (Xiang et al., 2013; Liang et al., 2014).

A meta-analysis indicated that at low temporal niche differentiation, LER decreased with increasing rates of nitrogen application (Yu et al., 2015). In the present study, LER decreased with the increasing use of nitrogen, consistent with previous studies. Xiao et al. (2018) found that in legume and cereal intercropping systems, the legume crop yields were not as stable as those of cereal crops. Using a meta-analysis, legume/grain intercropping was found to significantly increase grain yield only (Ren et al., 2016). The yield of grain in legume and grain intercropping systems should be increased as nitrogen nutrition provides an important contribution to increased grain yield in legume and grain intercropping systems. For the conditions in the present study, the yield of monocropped and intercropped wheat increased as nitrogen fertilizer was applied, the yield greatest at the N2 level, and decreasing at the N3 level. Zhao et al. (2012) found that the decrease in grain yield of winter wheat at high nitrogen levels (280 kg.ha^–1^) was due to the significant decrease in flag leaf photosynthetic rate, canopy photosynthetic rate, and crop growth rate at the filling stage. In the present study, the excessive use of nitrogen fertilizer was found to exacerbate the occurrence of wheat powdery mildew and stripe rust (Figures 2, 3). Serious disease outbreaks usually lead to yield loss. This demonstrates that yield loss caused by wheat disease is also a reason for the decrease in yield advantage in a wheat and faba bean intercropping systems.

A correlation analysis of the relationship between wheat disease index and yield was conducted (Figure 4). For different nitrogen application levels, the close correlation between wheat powdery mildew with yield and between rust and yield was not consistent for the two planting seasons. This is closely related to the effects of environmental factors (temperature, humidity) and nitrogen regulation on spore growth and biomass accumulation (Gómez-Rodrìguez et al., 2003; Guo et al., 2020). The temperature and humidity in the first planting season and at the wheat filling stage were lower, beneficial for the outbreak of rust spores compared with the higher temperature and humidity in the second planting season, in which an outbreak of powdery mildew spores appeared (Table 1 and Figures 2, 3). At the N1 level, the correlation between wheat disease index and yield reached a maximum, indicating that intercropping at the N1 level would improve grain yield by reducing disease index, while at the N2 and N3 levels, due to the severity of disease, the possibility of increasing crop yield through intercropping control measures is reduced (Figures 4A,B). For the 2-year comparison with monocropping, we found a close correlation between disease and yield in intercropping, indicating that intercropping was more effective than monocropping in controlling disease and increasing crop grain yield (Figure 4C) (Sahile et al., 2010). The most appropriate nitrogen level for the wheat and faba bean intercropping system was 180 kg⋅ha^–1^, as found at the N2 level. Therefore, nitrogen management in wheat and faba bean intercropping can balance the input and output of nitrogen and maximize the agro-ecosystem (Chen et al., 2013; Zhu et al., 2017b; Xiao et al., 2018).

In the present study, the effects of two common diseases and the yields of intercropped wheat for nitrogen management were further analyzed and expounded. The relationship between disease occurrence and yield was established. The contribution that intercropping makes to the occurrence of wheat disease and yield loss remains unknown. In future research studies, greater attention should be paid to quantitative examination of the ability of intercropping to recover yield losses caused by disease.

## Conclusion

Irrespective of the monocropping or intercropping regimen, the application of N tended to increase the occurrence and severity of wheat powdery mildew and stripe rust, the incidence and disease index of which was greatest at the N3 level. Wheat/faba bean intercropping effectively reduced the occurrence and degree of damage caused by two diseases of wheat, the severity of which varied by year and season, but was closely related to temperature and precipitation in a particular year. As application of N increased, the relative control effect of intercropping initially increased, then declined, with the control effect of intercropping at the N2 level found to be best. The correlation between disease index and yield indicated that appropriate nitrogen fertilizer regulation and other control measures combined with intercropping maximized disease control and provided the greatest intercropping yield increase. The present study provides the basis for the rational application of nitrogen fertilizer in wheat and faba bean intercropping, control of crop diseases, and crop yield improvement. In general terms, nitrogen fertilization with 180 kg.ha^−1^ was found to be optimal.

## Data Availability Statement

The original contributions presented in the study are included in the article/supplementary material, further inquiries can be directed to the corresponding author/s.

## Author Contributions

CL and LM conceived the original screening and research plans. CL finished writing and revising this thesis. KD and YD supervised the experiments and agreed to serve as the author responsible for contact and ensures communication. LM, JZ, and ZG provided technical assistance to CL. CL and LM designed the experiments. JZ and ZG analyzed the data. All authors contributed to the article and approved the submitted version.

## Conflict of Interest

The authors declare that the research was conducted in the absence of any commercial or financial relationships that could be construed as a potential conflict of interest.
